# Implementing the TRAPD model for the Saudi adaptation of the World Mental Health Composite International Diagnostic Interview 3.0

**DOI:** 10.1186/s13033-019-0267-x

**Published:** 2019-02-25

**Authors:** Mona Shahab, Feda Al‐Tuwaijri, Noha Kattan, Lisa Bilal, Sanaa Hyder, Zeina Mneimneh, Yu-chieh Lin, AbdulHameed Al-Habeeb, Abdullah Al-Subaie, Abdulrahman Binmuammar, Yasmin Altwaijri

**Affiliations:** 1 King Salman Center for Disability Research, Riyadh, Saudi Arabia; 2 Clinical Epidemiology, Leiden University Medical Center, Clinical Psychology, Leiden University, Leiden, The Netherlands; 3 Corporate Planning, Saudi Aramco, Dhahran, Saudi Arabia; 4 Vision Realization Office, General Sports Authority, Riyadh, Saudi Arabia; 5 Biostatistics, Epidemiology and Scientific Computing Department, King Faisal Specialist Hospital and Research Centre, MBC 03, PO Box 3354, Riyadh, 11211 Saudi Arabia; 6 SABIC Psychological Health Research & Applications Chair (SPHRAC), College of Medicine, King Saud University, Riyadh, Saudi Arabia; 7 Survey Research Center, Institute for Social Research, University of Michigan, Ann Arbor, MI USA; 8 Mental Health and Social Services, Ministry of Health, Riyadh, Saudi Arabia; 9 Edrak Medical Center, Riyadh, Saudi Arabia

**Keywords:** Translation, Mental health, Epidemiology, Survey methodology, Survey instrument

## Abstract

**Background:**

The World Mental Health-Composite International Diagnostic Interview (CIDI) 3.0, originally in English, is a fully-structured interview designed for the assessment of mental disorders. Although Arabic translations of CIDI from countries like Lebanon and Iraq exist, a Modern Standard Arabic translation was developed to suit the Saudi population. While the translation model used in the present paper has been used to translate instruments in Asian and European languages, there is no study to the best of our knowledge which has used this specific model to translate a validated instrument from English to Arabic.

**Case presentation:**

This paper describes the Saudi adaptation of CIDI 3.0. The TRAPD team translation model—comprising of translation, review, adjudication, pretesting and documentation—was implemented to carry out the Saudi adaptation of CIDI 3.0. Pretests involving cognitive interviewing and pilot study led to translation revisions which consequently confirmed that Saudi respondents had a good understanding of various items of the instrument. The adaptation procedures for the Saudi CIDI 3.0 were well documented and the instrument was linguistically validated with the Saudi population.

**Conclusion:**

The TRAPD model was successfully implemented to adapt the CIDI 3.0 to be used as the main survey instrument for the Saudi National Mental Health Survey, findings of which will provide health policy makers mental health indicators for health decision making and planning.

## Background

The Composite International Diagnostic Interview (CIDI) 3.0 was originally developed in English for the World Health Organization–World Mental Health (WHO–WMH) Survey Initiative. It is a comprehensive, fully-structured interview designed to be used by trained lay interviewers for the assessment of mental health disorders according to the definitions and criteria of Diagnostic and Statistical Manual (DSM) IV. The instrument is intended for use in epidemiological studies; and can measure the prevalence of mental disorders, the severity of these disorders, the burden associated with these disorders, and the barriers to treatment and service use [1].

The CIDI 3.0 has a number of sections including an introductory screening and lifetime review section, which is followed by several diagnostic sections assessing mood disorders, anxiety disorders, substance use disorders, childhood and other disorders. Additional sections assess functioning and physical comorbidity, treatment of mental disorders, risk factors, and socio-demographics. Literature has concluded that CIDI assesses disorders with acceptable reliability and validity [2–4].

Mental health disorders contribute significantly to the global burden of disease [5]. In the Kingdom of Saudi Arabia (KSA), mental health disorders are also a major public health concern [6]. To assess the magnitude of mental health problems in Saudi Arabia, the Kingdom joined the WMH Consortium and launched the Saudi National Mental Health Survey (SNMHS) using the CIDI 3.0. The SNMHS will consequently provide health policy makers with mental health indicators, which will be useful for health decision making and planning in KSA.

Arabic is a language spoken in various dialects. Even within a country like Saudi Arabia, the dialects differ from one region to another. However, Modern Standard Arabic is more commonly used throughout the country, where it is formally used in communication and uniformly appears in school texts and newspapers. Due to this, existing translations of CIDI from other Arabic-speaking countries like Lebanon [6] and Iraq [7] which have their own unique Arabic dialect were not used, and the CIDI 3.0 was translated and adapted to the Saudi culture through a series of steps. The Saudi version of the instrument may potentially benefit the Arab states of the Gulf Cooperation Council (GCC) as these Arab populations speak Arabic in a dialect, which is more familiar within the GCC than the Arabic spoken in Arab countries outside the GCC.

The main aim of this paper is to discuss the adaptation process of CIDI 3.0 for the SNMHS and its linguistic validation process. At the onset of the adaptation, changes were made to the English source sections, where some sections were entirely removed while others were modified. Once all sections were finalized, all new and revised sections were translated in Modern Standard Arabic.

## Case presentation

Few surveys have briefly discussed their instrument adaptation procedures under the WMH Survey Consortium, adapting the instrument in various languages such as Nepali and Spanish [8–10]. Moreover, while the translation model used in the present paper has been used to translate instruments in Asian languages [11] and European languages [12], there is no study to the best of our knowledge which used this specific model to translate a validated instrument from English to Arabic. This paper elaborates on the steps undertaken to adapt CIDI 3.0 for the SNMHS and its linguistic validation process. Prospective studies adapting their instruments should consider using similar rigorous methods employing robust translation frameworks and documentation of the involved processes.

## The TRAPD model

**Fig. 1:**
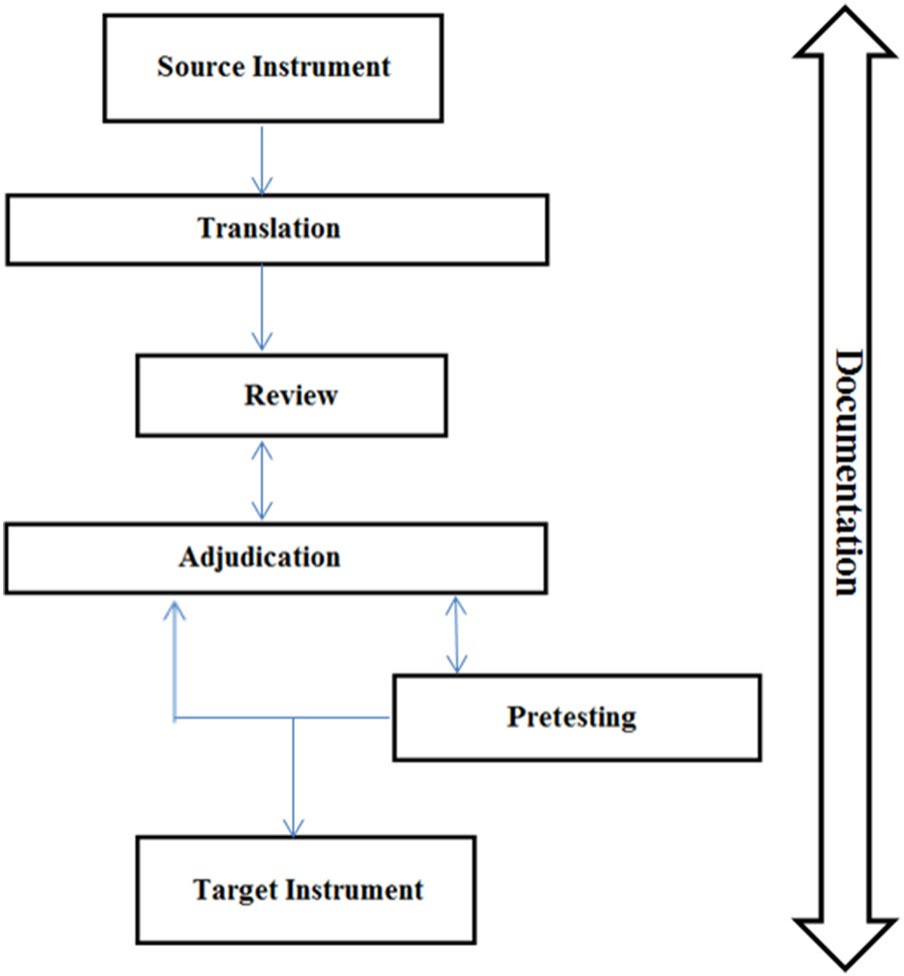
TRAPD team translation methodology (Adapted from Mohler et al. [19])

There are numerous approaches to health survey language translation methodology [13], which include forward translation, back-translation, using a team of translators and pretesting the translated instrument; yet there is no consensus on research standards to evaluate the quality of a translation. As this Arabic translation fell under the directive of the WMH Consortium, the SNMHS followed the WMH translation and adaptation guidelines as closely as possible [14]. At large, the TRAPD team translation model was implemented to carry out the Saudi adaptation of CIDI 3.0. The TRAPD model comprises of translation, review, adjudication, pretesting and documentation [15–18]. This model advocates for a team approach where a group of people with diverse expertise work together to produce an optimal version of the instrument, as the translation skill alone is not sufficient in a survey context [19]. The central aim of this translation approach is to focus on cultural equivalence rather than on literal equivalence at the level of words or entities referred to [14]. The translation and adaptation of the Saudi version of CIDI 3.0 was conducted between 2009 and 2010. Figure 1 outlines the steps used to produce the final Saudi translation using the TRAPD team translation model.

### Translation and review

Three sections (pharmacoepidemiology, gambling and personality disorder screen) from the original CIDI 3.0 were removed. Gambling was judged to be culturally inappropriate by the principal investigators as gambling is illegal in KSA; the other two sections were removed by our collaborators at Harvard University, who have extensively worked on the original instrument and are experts on the technicalities and logistics associated with the instrument. On the other hand, several sections were added to the Saudi instrument to measure aspects of the respondents’ lives that characterize the Saudi culture and that could play a role in the course of mental health disorders. These included religiosity, polygamy, dementia, disability, social satisfaction and attitude towards alcohol use. Religiosity and polygamy were regarded as important given that Islam is the main religion that drives the life of the Saudi population. The other sections were included as they formed the research interests of the SNMHS investigators. Dementia and disability are also significant to the population as for e.g. the size of older population continues to grow and thus, diseases linked with aging are on the rise [24]. Details of CIDI 3.0 sections unique to the SNMHS will be reported in the future along with the findings of the main survey.

The retained sections in their English source language were given to two native Saudi translators who divided the task between themselves to carry out a forward translation in Modern Standard Arabic. Following this, an initial review of the translation was carried out by two bilingual psychiatrists who read various sections together, and corrected or suggested replacement words, which they considered more suitable to convey the intended meaning of the original text. One of the principal investigators, who is a psychiatrist, then carried out a final review before moving on to the next stage. Since the recent translation literature [19] suggests that a back translated source provides only limited insight into the quality of the target language text, this step was not administered for the Saudi adaptation. However, team translation efforts made review and correction of the translation an intrinsic component of the adaptation process [14].

### Adjudication

Adjudication followed subsequently, in which the same principal investigator from the last stage as well as an expert panel consisting of psychiatrists, clinical psychologists, survey methodology experts, the other principal and co-investigators met with the original translators to review the entire translation again. Routine weekly meetings were held all year round with a different agenda every week. These meetings included collaborators from the WMH Coordinating Centers at Harvard University and University of Michigan, Ann Arbor, United States (US), and were led by the principal investigator(s), the project manager and the project coordinator. After these meetings, further modifications were made, based on consensus for suggested alternatives for words or expressions—see Table 1 for examples.

**Table 1:** Examples from the adjudication phase of the TRAPD model used to adapt Saudi CIDI 3.0

Question from the original CIDI 3.0	Modern Standard Arabic translation—before	Modern Standard Arabic translation—after	Explanation
PT10: Were you ever involved in a major natural disaster, like a devastating flood, hurricane, or earthquake?	هل سبق أن عايشت كارثة طبيعية هائلة ؟	هل سبق أن تعرضت لكارثة طبيعية هائلة؟	The Arabic word “عايشت” means “lived” as compared to the English word “involved”. It was therefore, changed to reflect the exact meaning of the question lived → involved
NSD9c: How often did you feel that everything was an effort?	تشعر بأن كل شيء يعتبر ثقيل عليك؟	تشعر بأن كل شيء تفعله يتطلب مجهود؟	The Arabic phrase “يعتبر ثقيل عليك” means “was heavy on you” as compared to the English phrase “was an effort”. It was changed to reflect the exact meaning of the question was heavy on you → was an effort
PD62c: How many professionals did you ever talk to about your attacks?	كم عدد المختصين على الإطلاق الذين تحدثت معهم حول هذه النوبات؟	كم عدد المختصين الذين تحدثت معهم في حياتك حول هذه النوبات؟	The Arabic phrase “على الإطلاق” means “at all” as compared to the English phrase “ever”. It was changed to reflect the exact meaning of the question at all → ever

Due to the sensitivity of some CAPI (Computer Assisted Personal Interview) sections (i.e. substance use, alcohol, polygamy, and religiosity) in general, and specifically within the Saudi culture, some sections were audio recorded. The Audio Computer Assisted Self Interview (ACASI) was gender specific to be more suited to the Saudi traditions and culture; a male respondent listened to the voice of a male interviewer, and a female respondent listened to the voice of a female interviewer. Once the voices were recorded, the Saudi team and the US teams worked together to make sure the voice files matched the text and the skips within ACASI were tested several times by different team members.

### Pretesting

The translated sections were pretested using 49 cognitive interviews. We used different cognitive probes such as probes for comprehension and probes for sensitivity across the whole response cycle to elicit respondent feedback—see Table 2 for examples. A purposive sample of Saudis was selected to participate in these cognitive interviews. It included both sexes of different age groups and educational backgrounds. The sample included both, participants with a history of mental disorders (49%) and those without such history (51%). The former group was selected from a local Saudi clinic in Riyadh based on their diagnosis of certain mental health disorders such as obsessive compulsive disorder, mania, generalized anxiety disorder (GAD), social phobia, panic disorder, depression, and bipolar disorder. Based on the findings of cognitive interviews, the translation was revised as appropriate, e.g. where there were discrepancies found between understanding of the item and its intended meaning. Further details of cognitive interviewing done for this translation can be found elsewhere [20].

**Table 2:** Cognitive interviewing: examples of probes for sensitivity and comprehension

Cognitive probes	Number of questions	Examples of probes
Sensitivity	137	1. Tell me more about your opinion for this question 2. How difficult is it to talk about this subject? 3. How easy or difficult is it to answer this question?
Comprehension	920	1. Can you use your own words and tell me what are we asking in this question? 2. Can you tell me what this question is asking about? 3. What does the phrase “…” mean to you in this question?
Total	1057	

### Pilot study

The revised instrument was then re-tested on a group of volunteers recruited by convenience sampling on the hospital premises (where the SNMHS team works), and subsequently modified again after the pilot study. The pilot study specifically employed a purposive sample of 190 households in Riyadh. Further details about the SNMHS pilot study can be found elsewhere [21]. Pilot feedback was obtained using debriefing forms which asked the respondents a list of questions including if they were able to understand the questions and if they found questions difficult to answer. This pretesting phase resulted in the removal of two sections in the instrument—neurasthenia and tobacco. This also helped to shorten the average interview length of the instrument, which during the pilot was found to be longer than expected (3.5 h) i.e. longer than the average length of the English version (approximately 2 h) [1]. Although the increased duration was partly attributed to the additional sections added to the Saudi CIDI 3.0, the culture-oriented routine of Saudi respondents contributed to the interview length (e.g. prayer breaks taken by respondents as Muslims pray 5 times a day). Thus, at the discretion of the principal investigators, these sections were removed. Following these changes, the adapted instrument for the main survey was launched.

### Documentation

All steps and stages of this translation were documented from start to finish. Project collaboration and management tools like e-room facilities, shared drives and shared documents accessible to the entire translation team were used to share updates, edits and comments. Since documentation of each step is used as a quality assurance and monitoring tool [19], this aspect helped the present adaptation in terms of attaining high quality standards. Each stage of the team translation process was built on the foregoing steps and used the documentation from the previous step to inform the next [19]. Systematic documentation also allowed for easy comparison between the various versions produced for the translated sections.

## Discussion

It is important to note that even after the launch of the main survey, some modifications were made to the translation of Saudi CIDI 3.0, which were again documented. For instance in 2013, three sections (Specific Phobia, Social Networks and Oppositional Defiant Disorder) were removed to further shorten the main interview length to make it comparable to the interview length of the English version.

Overall, more than 40 sections of the original CIDI 3.0 with over 500 pages were translated to Modern Standard Arabic for the Saudi adaptation. The number of translated questions in each section varied—some sections, such as the post-traumatic stress disorder section, comprise of up to 300 questions whereas other sections like mania, obsessive compulsive disorders or illegal drug use have around 70 questions. Furthermore, substantial time was invested in programming the Arabic CIDI 3.0. The Saudi team worked closely with the programmers in the US due to the change in word order/sequence when Arabic text was inserted in Notepad++ v6.6.1, a free source code editor that supports different programming languages. According to a conservative estimate, the whole translation process took a total of 1038 h. Table 3 shows an outline of the Saudi CIDI 3.0 with its final sections.

**Table 3:** Sections in the Saudi version of CIDI 3.0

I.	Screening and lifetime review	
II.	Disorders	
	Mood	Major depression, mania
	Anxiety	Panic disorder, social phobia, agoraphobia, GAD, post-traumatic stress disorder, obsessive–compulsive disorder
	Substance abuse	Alcohol dependence, illegal substance use
	Childhood	Attention-deficit/hyperactivity disorder, conduct disorder, separation anxiety disorder
	Other	Intermittent explosive disorder, premenstrual disorder, psychosis screen, eating disorder
III.	Functioning and physical disorders	Suicidality, 30-day functioning, 30-day symptoms, physical comorbidity
IV.	Treatment	Services
V.	Risk factors	Personality, social satisfaction, childhood experiences, family burden
VII.	Sociodemographics	Employment, finances, marriage, children, demographics
VII.	Country-specific sections	Attitude towards alcohol use, religiosity, polygamy, disability, dementia, disability burden

The adapted CIDI 3.0 has been linguistically validated with the Arabic-speaking Saudi population in KSA. Following a rigorous multi-step translation methodology, which incorporated the input of translators and other experts both at the national and international level, and included pretesting to assess understandability of the various sections, the Saudi version of CIDI 3.0 was considered conceptually equivalent to the English source. The translation and evaluation methods employed by the SNMHS supported a collaborative research environment. As evidenced thus, the project benefited from the diverse expertise of individual team members, who in turn benefitted from the experience of the process. The SNMHS is now sought after by other projects in KSA for its experience relating to various aspects including translation.

Although it was a time-consuming and arduous process (especially because of the length and size of the instrument), the duration was well utilized as the chief concern of the SNMHS team was to (i) use an evidence-based framework to translate the instrument, and (ii) make it accessible to the Saudi population. Consequently, the SNMHS was one of the pioneering surveys in Saudi Arabia to employ a meticulously systematic approach, which facilitated the successful translation and adaptation of a comprehensive instrument. Psychometric validation may be a useful next step for verifying the validity of the adapted instrument, as the survey did not undertake a calibration study. Nonetheless, literature indicates that testing for psychometric properties, albeit highly recommended, is not required for approval of a translated version of a questionnaire [22]. Future research should also consider investigating the effects of specific words/phrases used in the translated instrument among various Arab populations. Moreover, prospective studies should consider exploring the effects of implementation design features such as the mode used (e.g. CAPI vs. ACASI).

The CIDI 3.0 translated in Modern Standard Arabic is potentially a valuable resource for researchers interested in examining mental health issues in the Arab states of the GCC. Despite the fact that the scale was linguistically validated in a Saudi population, it could suit other Arab populations, particularly those from the GCC; further studies using the Saudi version of CIDI 3.0 are needed to confirm this assumption. On the whole, we have shared our work to benefit prospective studies, given that determining how well a translation methodology succeeds in providing a culturally responsive and linguistically appropriate measure of health across cultures is critical for prospective research aimed at investigating health disparities [23].

## Abbreviations

CIDI: Composite International Diagnostic Interview
WHO: World Health Organization
WMH: World Mental Health
DSM: Diagnostic and Statistical Manual
KSA: Kingdom of Saudi Arabia
SNMHS: Saudi National Mental Health Survey
TRAPD: translation, review, adjudication, pretesting and documentation
CAPI: Computer Assisted Personal Interview
ACASI: Audio Computer Assisted Self Interview
US: United States
GCC: Gulf Cooperation Council
GAD: Generalized Anxiety Disorder
